# Prevalence and Risk Factors of Carotid Artery Stenosis (CAS) Among Cardiac Surgery Patients

**DOI:** 10.7759/cureus.37634

**Published:** 2023-04-16

**Authors:** Danah K Alsalmi, Rawan Abdeen

**Affiliations:** 1 Faculty of Applied Medical Sciences, Radiologic Sciences, King Abdulaziz University, Jeddah, SAU

**Keywords:** ultrasound, risk factor, cardiac surgery, atherosclerosis, carotid artery stenosis, prevalence

## Abstract

Introduction: Cardiac disease and carotid atherosclerosis rates have increased in recent years. Carotid artery stenosis (CAS) has been recognized as a high-risk factor of perioperative stroke among cardiac surgery patients.

Aims: The aims of the study are to identify the prevalence and common risk factors of CAS among patients undergoing cardiac surgery that include coronary artery bypass surgery or valvular cardiac surgery.

Materials and Methods: This retrospective cross-sectional study was conducted in the radiology department at Medina Cardiac Center, Al Madinah Al-Munawara. The inclusion criteria for the study were patients aged ≥ 20 years who were scheduled for coronary artery bypass surgery or valvular cardiac surgery and had carotid duplex examination before surgery. A Philips X matrix IU22 linear-array ultrasound probe (Philips, Bothell, WA) was used to scan the common carotid artery (CCA), internal carotid artery (ICA), external carotid artery (ECA), and vertebral artery.

Results: Of the 261 patients in this study, 78.5% (*n* = 205) were male. The mean age of patients was 61.6 ± 11.3 years (median: 62.0; range: 55.5-68.0). The overall prevalence of CAS was 71% (*n* = 187): 52% (*n* = 136) with bilateral CAS and 19.5% (*n* = 51) with unilateral CAS. Age group was significantly associated with bilateral CAS and the severity of CAS (p* *= 0.001). Diabetes mellitus, hypertension and both diabetes mellitus and hypertension together were significantly associated with CAS status (p* *< 0.05, for all). A significantly higher proportion of smokers had a mild level of CAS on the left side compared to non-smokers (55.8% vs. 46.5%, p* *= 0.033). Gender and weight status were not linked to severity of CAS.

Conclusion: This study shows a high prevalence of CAS among cardiac surgery patients. In addition, older age, diabetes mellitus, and hypertension were found to be major risk factors for CAS. Gender and weight status were not associated with CAS. Preoperative carotid duplex scan is a useful exam to identify CAS among cardiac surgery patients and, therefore, to predict and reduce postoperative neurological complications.

## Introduction

 The term atherosclerosis refers to a biological event characterized by disease of the vascular intima layer that can potentially affect the entire arterial system from the aorta to the coronary arteries [1]. The term atherosclerosis is derived from the Greek words atherosis (meaning an accumulation of fat in the core of the plaque) and sclerosis (which describes the thickening of the intima layer of the arterial wall) [2]. Atherosclerosis is a common disease that is characterized by fatty deposits called intimal plaques. The development of these plaques starts with the deposition of cholesterol crystals in the intimal layer. The plaque then grows as apoptotic cells and smooth muscle accumulates to form a bulge inside of the vessels, which consequently decreases blood flow. This bulge then accumulates calcium and additional dead cells inside the lesion, which causes the hardening of the artery. A ruptured plaque will lead to the obstruction of blood flow [3]. Atherosclerosis is the most common underlying cause of coronary, peripheral, and carotid artery disease. According to reports, atherosclerosis alone is rarely lethal, unless the atherosclerotic plaque is ruptured and thrombosed, which will lead to life-threatening clinical events, such as acute coronary syndrome and stroke [4-5]. Atherosclerosis is a systematic inflammatory disease and, therefore, a strong relation between coronary atherosclerosis and carotid artery stenosis (CAS) has commonly been assumed [6-7]. Moreover, there is evidence to suggest that significant CAS is common among patients undergoing cardiac surgery with a high risk of critical neurological events [8-9]. Approximately 30% of annual global mortality is attributable to cardiovascular disease, while an additional 10% is attributable to cerebrovascular events [10-11].

According to Vranic (2017), the factors that play a major role in the pathogenesis and prediction of treatment for cardiovascular and cerebrovascular diseases are: hyperlipidemia, diabetes mellitus, hypertension, and smoking [12]. It has been reported that patients undergoing coronary bypass surgery usually have atherosclerosis in the carotid artery. Therefore, significant CAS is a critical cause of strokes among patients with heart disease, who have undergone cardiac revascularization surgery [13-14]. In Western countries, the prevalence of CAS among heart surgery patients in recent decades has risen from 12.8% to 22% [10-11]. It is believed that the extent of coronary artery illness is directly related to the severity of CAS. Zhang et al. (2015) hold the view that around 20% of patients suffering from multivessel coronary artery disease had significant CAS [15]. Several potential risk factors for CAS have been demonstrated, such as hypertension, smoking, diabetes, age, gender, peripheral vascular disease, and coronary artery disease [12, 15]. According to the World Health Organization (‎2018), there is a higher prevalence of diabetes mellitus, being overweight, and smoking among the Saudi population [16], thus exposing them to a higher risk of atherosclerosis. Furthermore, very few studies, including those conducted on the Saudi population, have assessed the preponderance of CAS among cardiac surgery patients [17]. Therefore, the primary aim of this study is to identify the prevalence of CAS among cardiac surgery patients, with the secondary aim of determining the common risk factors of CAS among patients undergoing heart surgery in Madinah Cardiac Center.

## Materials and methods

A retrospective cross-sectional study was conducted in the radiology department at Medina Cardiac Center in Al Madinah Al-Munawara between February 2021 and August 2022. The inclusion criteria for this study were patients aged ≥ 20 years who were scheduled for coronary artery bypass surgery or valvular cardiac surgery and underwent a carotid duplex examination before surgery. The study was approved by the Scientific and Health Research Ethics Committee in Madinah. This study excluded patients under 20 years old who did not undergo heart surgery or who had heart surgery without undergoing a carotid examination. All patients underwent a carotid duplex examination using a Philips X matrix IU22 linear-array ultrasound probe with a frequency of 9-3 MHz. All ultrasound examinations were performed by an experienced radiologist. Patients were examined in the supine position with their head slightly tilted to the side. The carotid vascular preset was activated on the ultrasound machine. The scanning process began with a B-mode transverse view of the common carotid artery (CCA) from its proximal segment to its bifurcation into the internal carotid artery (ICA) and external carotid artery (ECA) as high in the neck as possible and then continued in the longitudinal view. The carotid arteries were scanned to assess anatomical variation and abnormal findings, which include increased intima media thickness, plaque or calcification, stenosis, and occlusion.

Ultrasound color doppler was used to detect the direction of blood flow, filling of blood flow in the vascular lumen, aliasing artifacts, and the status of blood flow. Moreover, spectral analysis was employed to measure the following blood flow velocities: peak systolic velocity (PSV), end diastolic velocity (EDV), and ICA/CCA PSV ratio. The severity of CAS was calculated by measuring the patent lumen at the stenotic site (diameter redaction), PSV, EDV, and ICA/CCA PSV ratio. The vertebral artery was also scanned during the carotid examination. Patients were classified into groups according to the level of severity. This classification was based on the diagnostic criteria of the Society of Radiologists in Ultrasound Consensus Conference in Table 1 [18].

**Table 1:** Severity classification of CAS. ICA, internal carotid artery; PSV, peak systolic velocity; EDV, end diastolic velocity; CCA, common carotid artery; CAS, carotid artery stenosis

	Normal	Mild	Moderate	Severe	Near occlusion	Total occlusion
Plaque estimate (%)	No plaque No intimal thickening	Visible <50	Visible ≥50	Visible ≥50 Clear luminal stenosis	Visible nearly occupying whole lumen	Visible No patent lumen
Stenosis (%)	No	<50	50-69	>70	>90	100
ICA PSV (cm/s)	<125	<125	125-230	>230	>>230	
ICA EDV (cm/s)	<40	<40	40-100	>100	>100	
ICA / CCA ratio	<2	<2	2-4	>4	>4	

Statistical analysis 

Suitable purposive sampling with a standardized datasheet (Microsoft ExcelTM, Microsoft Corporation, One Microsoft Way, Redmond, WA) was utilized to collect crucial information on the study population. Data in this study were analyzed using IBM SPSS Statistics for Windows (Version 20.0; IBM Corp., Armonk, NY). Descriptive data for categorical variables were presented as frequencies (percentages). The normality of the distribution of continuous variables [age in years; body mass index (BMI) in kg/m2; ICA/CCA PSV ratio (right and left)] was assessed using the Shapiro-Wilk test and all variables were skewed (p < 0.05). Data concerning age and BMI were presented as the mean ± standard deviation (SD) and median (interquartile range). Fisher’s exact test was used to assess the link between two categorical variables and post-hoc tests were used to further investigate significant findings. The Kruskal-Wallis test was used to compare median age and BMI across the different groups (patients with and without CAS) and pairwise comparisons were conducted to further explore the associations found. Multinomial logistic regression analysis was performed to investigate determinants of bilateral and unilateral CAS. Variables were coded as follows: gender (Female = 0, Male = 1); age group (≤ 50 years = 1, 51-60 years = 2, 61-70 years = 3, > 70 years = 4); diabetes (No diabetes = 0, Diabetes = 1); hypertension (No hypertension = 0, Hypertension = 1); diabetes and hypertension (No = 0, Yes = 1); smoking status (Non-smoker = 0, Smoker = 1); weight status (Underweight = 1, Healthy weight = 2, Overweight = 3, Obese = 4); and severity of CAS (Normal = 0, Mild = 1, Moderate = 2, Severe = 3, Total occlusion = 4). All tests used were two-tailed and with a significance of 95%.

## Results

Sample characteristics

The total number of patients included in this study was 261 and 78.5% (n = 205) were male. The mean age of patients was 61.6 ± 11.3 years (median: 62.0; range: 55.5-68.0), with 57.9% (n = 151) > 60 years old. Forty percent of the study sample were smokers (n = 104). The mean BMI of patients was 29.0 ± 5.44 kg/m2 (median: 27.7; range 25.4-32.1), with 79.3% (n = 207) being overweight or obese. Diabetes mellitus and hypertension were similarly prevalent (71.3% and 72.8%, respectively), and 59.8% (n = 156) of patients had both diabetes mellitus and hypertension. The characteristics of the study sample are presented in Table 2. 

**Table 2:** Sample characteristics (n= 261). BMI, body mass index

Variable	n	%
Sex		
Male	205	78.5
Female	56	21.5
Age group		
≤ 50 years	42	16.1
51-60 years	68	26.1
61-70 years	105	40.2
> 70 years	46	17.6
Smoking status		
Smoker	104	39.8
Non-smoker	157	60.2
Weight status		
Underweight (BMI < 18.5 kg/m^2^)	1	0.40
Healthy weight (BMI 18.5-24.9 kg/m^2^)	53	20.3
Overweight (BMI 25.0-29.9 kg/m^2^)	117	44.8
Obese (BMI ≥ 30.0 kg/m^2^)	90	34.5
Chronic diseases		
Diabetes	186	71.3
Hypertension	190	72.8
Diabetes and hypertension	156	59.8

Description of clinical data

Descriptive clinical data linked to bilateral and unilateral CAS among the study sample are shown in Table 3. Among the study sample: 61% (n = 160) have increased intima media thickness of the right carotid artery and 64.0% (n = 167) have increased intima media thickness of the left carotid artery; plaque of the right carotid artery was reported among 59.8% (n = 156) and plaque of the left carotid artery among 64.0% (n = 167); and the mean right side ICA/CCA PSV ratio was 1.96 ± 8.65 (median: 1.06; range: 0.89-1.30) and the mean left side ICA/CCA PSV ratio was 1.67 ± 6.17 (median: 1.10; range: 0.90-1.35). The severity of right and left CAS was assessed and about half of the sample reported a mild level (< 50). Abnormalities in the right and left vertebral artery were reported among 1.10% (n = 3) of the patients included in this study. 

**Table 3:** Descriptive clinical data linked to bilateral and unilateral carotid stenosis among the study sample (n= 261).

	Right side	Left side
Intima media thickness of carotid artery		
Normal	101 (38.7)	94 (36.0)
Increased	160 (61.3)	167 (64.0)
Plaque of carotid artery		
No PLAQUE	105 (40.2)	94 (36.0)
PLAQUE	156 (59.8)	167 (64.0)
Severity of carotid stenosis		
Normal (zero)	104 (39.8)	94 (36.0)
Mild (< 50)	127 (48.7)	131 (50.2)
Moderate (50-69)	26 (10.0)	30 (11.5)
Severe (> 70)	2 (0.08)	3 (1.10)
Near occlusion (> 90)	0 (0.00)	2 (0.77)
Total occlusion (100)	2 (0.08)	1 (0.38)
Vertebral artery abnormalities		
Normal	251 (96.2)	253 (96.9)
Abnormal	3 (1.10)	3 (1.10)
Cannot be assessed	7 (2.70)	5 (1.90)

Prevalence of bilateral and unilateral CAS and association with characteristics of the study sample

**Figure 1:**
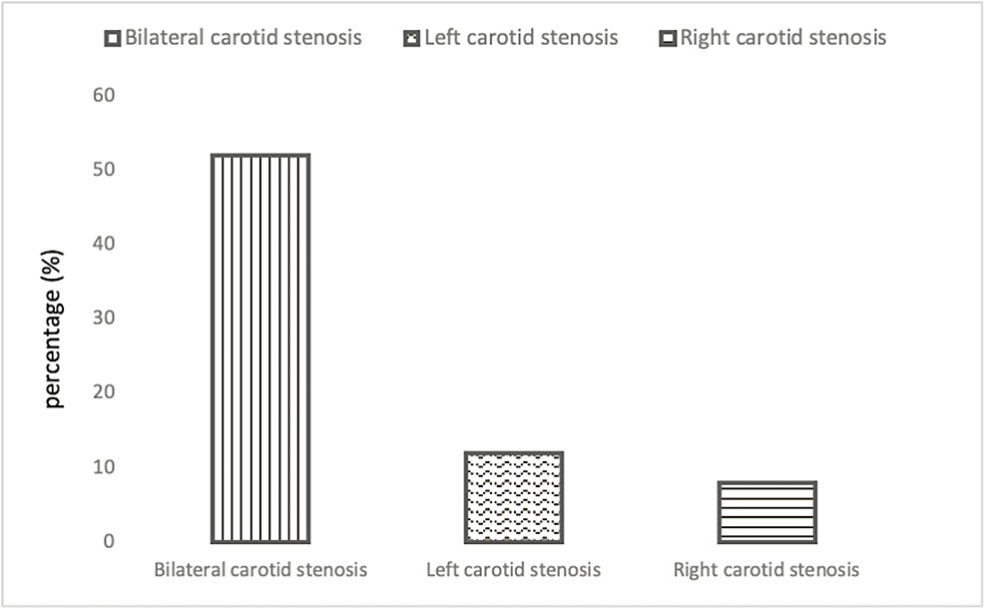
Prevalence of bilateral and unilateral carotid stenosis among the study sample.

The prevalence of CAS among the study population was 71% (n = 187): 52% (n = 136) with bilateral CAS and 19.5% (n = 51) with unilateral CAS. Over half of the patients included in this study had bilateral CAS (n = 136) whereas 7.70% (n = 20) had right unilateral CAS and 11.9% (n = 31) had left unilateral CAS (see Figure 1). 

The characteristics of the study sample and associations with bilateral and unilateral CAS are shown in Table 4. Gender was not linked to bilateral or unilateral CAS status (p = 0.259) but age group was significantly associated (p = 0.001). Furthermore, age in years was significantly associated with bilateral and unilateral CAS status (p < 0.001); 83 (79.1%) patients were aged 61-70 years and 47 (69.2%) patients were aged 51-60 years. Results of the pairwise comparison show that median age was significantly higher among patients with bilateral CAS compared to participants with normal carotid artery [64.0 (59.0-70.0) vs. 58.0 (48.0-64.3) years, respectively; p < 0.001]. Smoking status and weight status were not associated with bilateral or unilateral CAS status (p = 0.681 and p = 0.562, respectively). Median BMI was similar across the different groups: normal carotid artery, 28.4 (26.1-32.1) kg/m2; bilateral CAS, 27.6 (25.0--32.3) kg/m2; right CAS, 26.8 (24.0-30.2) kg/m2; left CAS, 29.3 (26.1-32.9) kg/m2 (p = 0.190). Diabetes mellitus, hypertension, and both diabetes mellitus and hypertension were significantly associated with bilateral and unilateral CAS status (p < 0.05, for all). 

**Table 4:** Characteristics of the study sample and associations with bilateral and unilateral carotid stenosis (n=261). *Significant at the 95% confidence level. Numbers presented in table are frequencies (%). Data presented in table were obtained using Fisher’s Exact test.

	Normal carotid artery (n= 74)	Bilateral carotid stenosis (n= 136)	Right carotid stenosis (n= 20)	Left carotid stenosis (n= 31)	p-value
Sex					
Male	52 (25.4)	111 (54.1)	17 (8.30)	25 (12.2)	0.259
Female	22 (39.3)	25 (44.6)	3 (5.40)	6 (10.7)	
Age group					
≤ 50 years	23 (54.8)	10 (23.8)	5 (11.9)	4 (9.50)	0.001*
51-60 years	21 (30.9)	32 (47.1)	7 (10.3)	8 (11.8)	
61-70 years	22 (21.0)	63 (60.0)	5 (4.80)	15 (14.3)	
> 70 years	8 (17.4)	31 (67.4)	3 (6.50)	4 (8.70)	
Smoking status					
Non-smoker	46 (29.3)	84 (53.5)	10 (6.40)	17 (10.8)	0.681
Smoker	28 (26.9)	52 (50.0)	10 (9.60)	14 (13.5)	
Weight status					
Underweight	0 (0.00)	1 (100)	0 (0.00)	0 (0.00)	0.562
Healthy weight	10 (18.9)	32 (60.4)	5 (9.40)	6 (11.3)	
Overweight	38 (32.5)	58 (49.6)	10 (8.50)	11 (9.40)	
Obese	26 (28.9)	45 (50.0)	5 (5.60)	14 (15.6)	
Diabetes mellitus					
No diabetes	39 (52.0)	24 (32.0)	7 (9.30)	5 (6.70)	<0.001*
Diabetes	35 (18.8)	112 (60.2)	13 (7.00)	26 (14.0)	
Hypertension					
No hypertension	32 (45.1)	24 (33.8)	6 (8.50)	9 (12.7)	0.001*
Hypertension	42 (22.1)	112 (58.9)	14 (7.40)	22 (11.6)	
Diabetes mellitus and hypertension					
No	46 (43.8)	37 (35.2)	10 (9.50)	12 (11.4)	<0.001*
Yes	28 (17.9)	99 (63.5)	10 (6.40)	19 (12.2)	

Determinants of bilateral and unilateral CAS

Results obtained from the logistic regression analysis that aim to investigate determinants of bilateral and unilateral CAS are shown in Table 5. Gender, smoking status, and weight status did not determine bilateral and unilateral CAS among the patients included in this study. However, patients aged ≤ 50 years have significantly lower odds of having bilateral CAS compared to patients aged > 70 years [odds ratio (OR) = 0.11, 95% confidence Interval (CI) = 0.04-0.33, p < 0.001]. In addition, patients with no diabetes have significantly lower odds of having bilateral and left CAS compared to diabetic patients (OR = 0.19, 95% CI = 0.10-0.36, p < 0.001 and OR = 0.17, 95% CI = 0.06-0.50, p = 0.001, respectively). Patients with no hypertension have significantly lower odds of having bilateral CAS compared to patients with hypertension (OR = 0.28, 95% CI = 0.15-0.53, p < 0.001). Patients with no diabetes or hypertension (or both) have significantly lower odds of having bilateral and left CAS compared to patients with both diabetes and hypertension together (OR = 0.23, 95% CI = 0.13-0.42, p < 0.001 and OR = 0.38, 95% CI = 0.16-0.91, p= 0.030, respectively). 

**Table 5:** Determinants of bilateral and unilateral carotid stenosis. *Significant at the 95% confidence level. One underweight patient was eliminated from the analysis to avoid error in output. Reference category for outcome in all models was “normal carotid artery”

Variables	Odd ratio	95% Confidence interval	p-value
Sex			
Bilateral carotid stenosis	0.53	0.28-1.03	0.062
Right carotid stenosis	0.42	0.11-1.57	0.196
Left carotid stenosis	0.57	0.20-1.58	0.276
Age group			
Bilateral carotid stenosis			
≤ 50 years	0.11	0.04-0.33	< 0.001*
51-60 years	0.39	0.15-1.02	0.055
61-70 years	0.74	0.30-1.85	0.518
> 70 years	Reference category		
Right carotid stenosis			
≤ 50 years	0.58	0.11-3.00	0.515
51-60 years	0.89	0.18-4.31	0.884
61-70 years	0.61	0.12-3.14	0.551
> 70 years	Reference category		
Left carotid stenosis			
≤ 50 years	0.35	0.07-1.73	0.196
51-60 years	0.76	0.18-3.25	0.713
61-70 years	1.36	0.35-5.36	0.657
> 70 years	Reference category		
Smoking status			
Bilateral carotid stenosis	0.98	0.55-1.76	0.955
Right carotid stenosis	0.61	0.23-1.65	0.328
Left carotid stenosis	0.74	0.32-1.73	0.485
Weight status			
Bilateral carotid stenosis			
Healthy weight	1.85	0.78-4.36	0.161
Overweight	0.88	0.47-1.66	0.697
Obese	Reference Category		
Right carotid stenosis			
Healthy weight	2.60	0.62-11.0	0.193
Overweight	1.37	0.42-4.47	0.604
Obese	Reference category		
Left carotid stenosis			
Healthy weight	1.11	0.34-3.71	0.860
Overweight	0.54	0.21-1.37	0.193
Obese	Reference category		
Diabetes mellitus			
Bilateral carotid stenosis	0.19	0.10-0.36	< 0.001*
Right carotid stenosis	0.48	0.17-1.35	0.165
Left carotid stenosis	0.17	0.06-0.50	0.001*
Hypertension			
Bilateral carotid stenosis	0.28	0.15-0.53	< 0.001*
Right carotid stenosis	0.56	0.20-1.63	0.288
Left carotid stenosis	0.54	0.22-1.32	0.176
Diabetes mellitus and hypertension			
Bilateral carotid stenosis	0.23	0.13-0.42	< 0.001*
Right carotid stenosis	0.61	0.23-1.65	0.328
Left carotid stenosis	0.38	0.16-0.91	0.030*

Association between sample characteristics and severity of CAS

**Figure 2:**
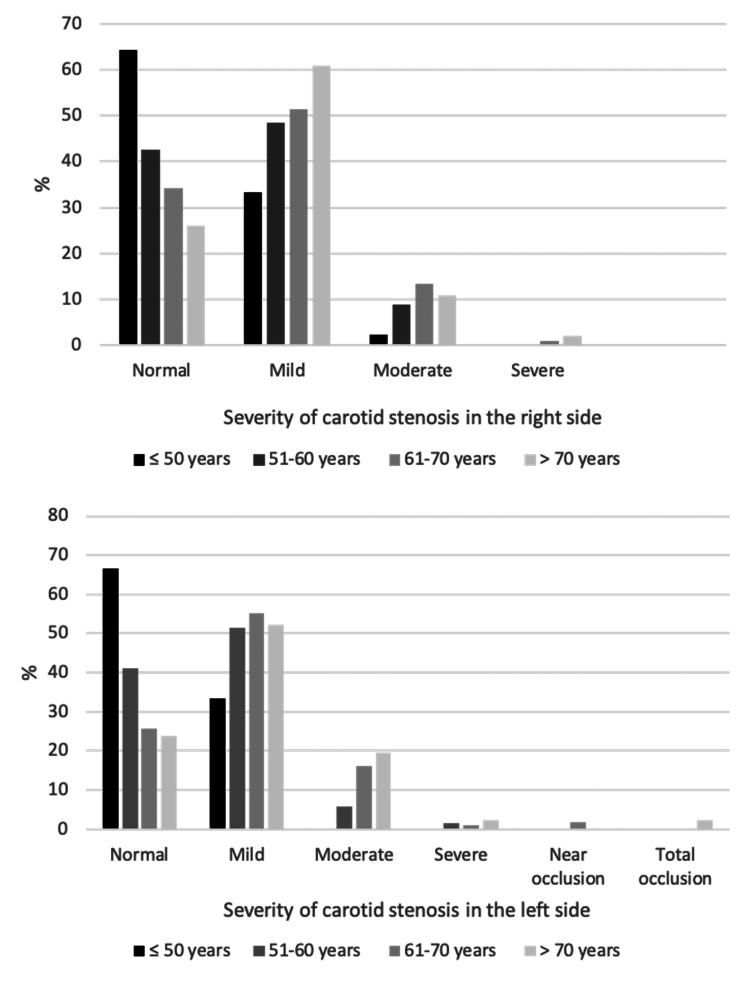
Association between age groups and severity of carotid stenosis in the right and left sides.

**Figure 3:**
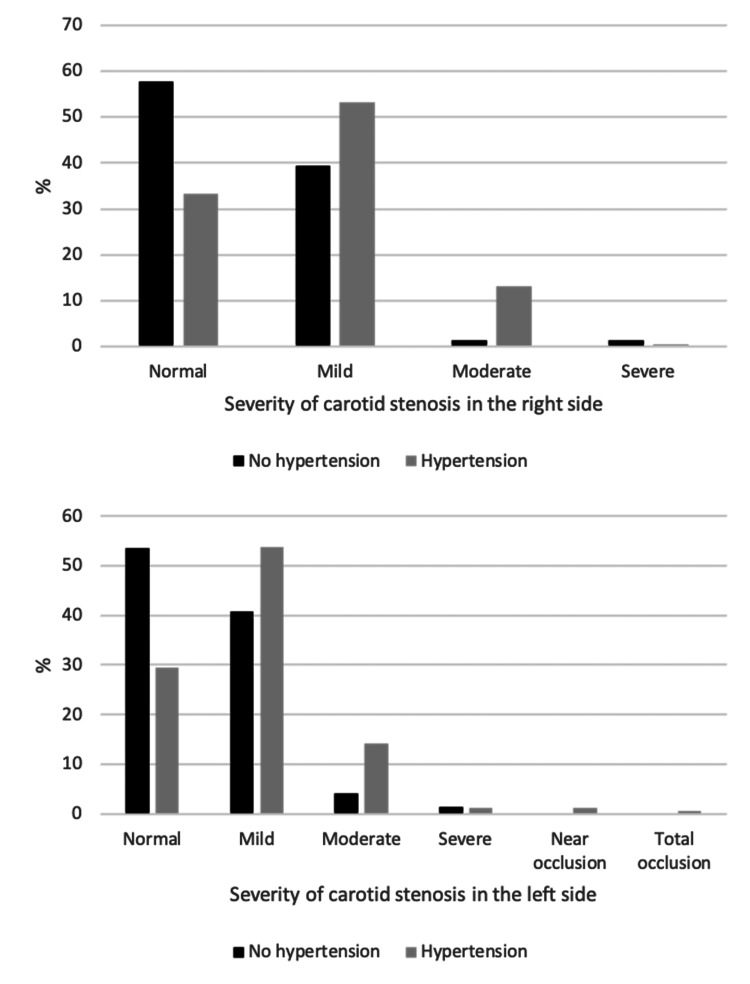
Association between hypertension and severity of carotid stenosis in the right and left sides.

**Figure 4:**
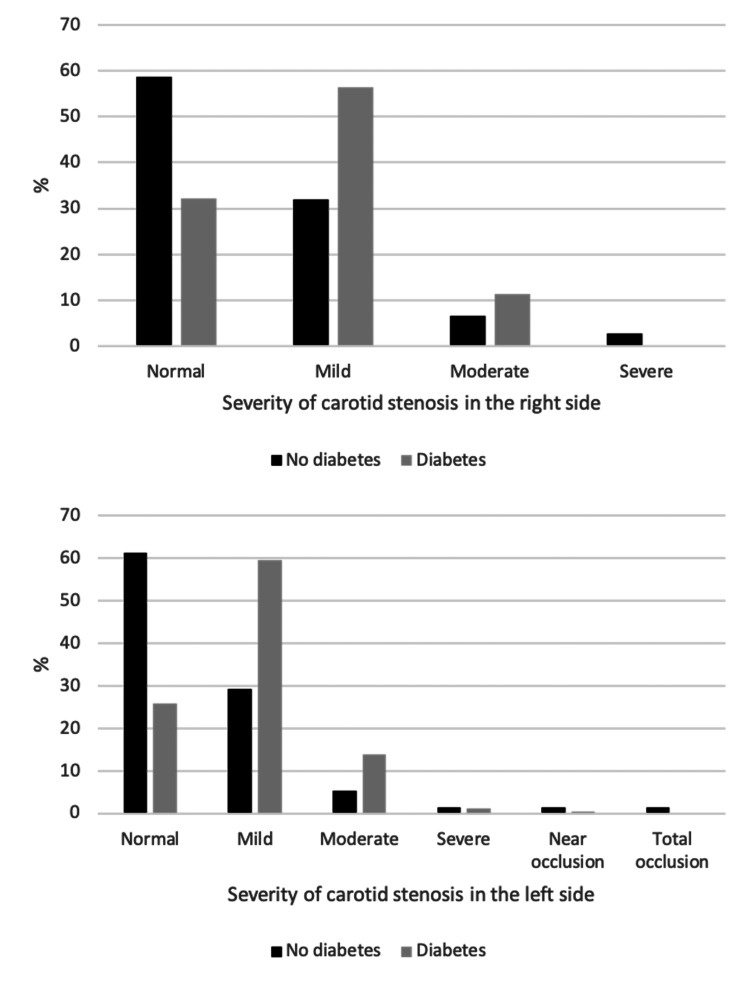
Association between diabetes and severity of carotid stenosis in the right and left sides.

**Figure 5:**
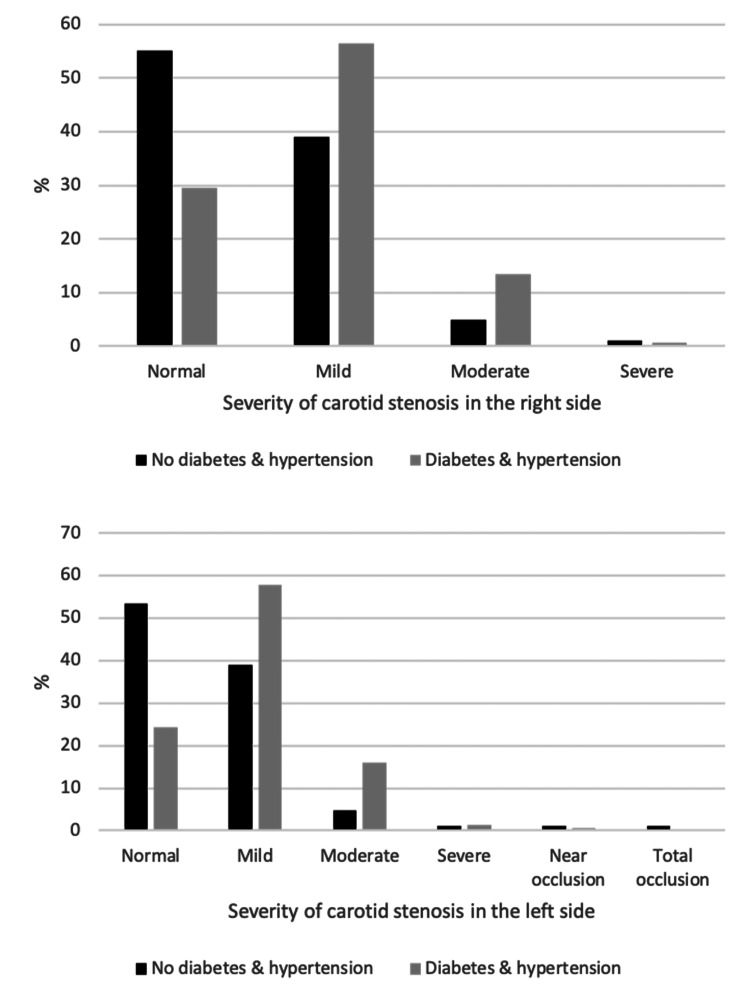
Association between both diabetes and hypertension and severity of carotid stenosis in the right and left sides.

**Figure 6:**
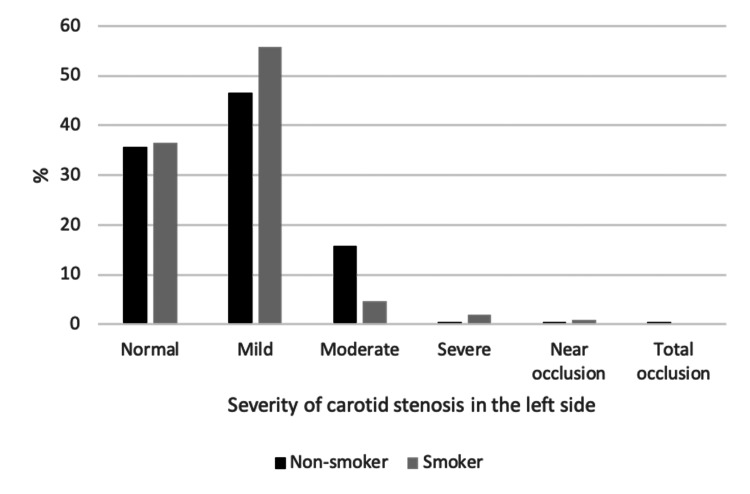
Association between smoker and severity of the left carotid stenosis among the study sample.

The associations between sample characteristics and severity of CAS among the study sample are shown in Table 6 , Figures 2-6. Gender and weight status were not linked to severity of CAS, whereas age group was linked to bilateral CAS, smoking status to left CAS and having diabetes, hypertension or both diseases was linked to bilateral CAS. A significantly higher proportion of patients aged > 70 years had a mild level of CAS on the right side compared to the other groups (p = 0.015), whereas a significantly higher proportion of patients aged 61-70 years had a mild level of CAS on the left side compared to the other groups (p < 0.001). A significantly higher proportion of smokers had a mild level of CAS on the left side compared to non-smokers (55.8% vs. 46.5%, p = 0.033). Furthermore, a significantly higher proportion of patients with diabetes, hypertension or both diseases had a mild level of CAS in the right and left sides compared to patients with no diabetes, hypertension or both diseases (p < 0.05, for all). 

**Table 6:** Association between sample characteristics and severity of carotid stenosis among the study sample. *Numbers presented in the table are frequencies (%). Data presented in the table were obtained using Fisher’s Exact test. *Significant at the 95% confidence level

Variables	Severity in right side				Severity in left side					
	Normal carotid artery	Mild	Moderate	Severe	Normal carotid artery	Mild	Moderate	Severe	Near occlusion	Total occlusion
Sex										
Male	28 (50.0)	25 (44.6)	3 (5.40)	0 (0.00)	25 (44.6)	22 (39.3)	7 (12.5)	1 (1.80)	1 (1.80)	0 (0.00)
Female	76 (37.1)	104 (50.7)	23 (11.2)	2 (1.00)	69 (33.7)	109 (53.2)	23 (11.2)	2 (1.00)	1 (0.50)	1 (0.50)
p-value	0.257				0.278					
Age group										
≤ 50 years	27 (64.3)	14 (33.3)	1 (2.40)	0 (0.00)	28 (66.7)	14 (33.3)	0 (0.00)	0 (0.00)	0 (0.00)	0 (0.00)
51-60 years	29 (42.6)	33 (48.5)	6 (8.80)	0 (0.00)	28 (41.2)	35 (51.5)	4 (5.90)	1 (1.50)	0 (0.00)	0 (0.00)
61-70 years	6 (34.3)	54 (51.4)	14 (13.3)	1 (1.00)	27 (25.7)	58 (55.2)	17 (16.2)	1 (1.00)	2 (1.90)	0 (0.00)
> 70 years	12 (26.1)	28 (60.9)	5 (10.9)	1 (2.20)	11 (23.9)	24 (52.2)	9 (19.6)	1 (2.20)	0 (0.00)	1 (2.20)
p-value	0.015*				<0.001*					
Smoking status										
Non-smoker	62 (39.5)	76 (48.4)	18 (11.5)	1 (0.60)	56 (35.7)	73 (46.5)	25 (15.9)	1 (0.60)	1 (0.60)	1 (0.60)
Smoker	42 (40.4)	53 (51.0)	8 (7.70)	1 (1.00)	38 (36.5)	58 (55.8)	5 (4.80)	2 (1.90)	1 (1.00)	0 (0.00)
p-value	0.773				0.033*					
Weight status										
Underweight	0 (0.00)	0 (0.00)	1 (100)	0 (0.00)	0 (0.00)	1 (100)	0 (0.00)	0 (0.00)	0 (0.00)	0 (0.00)
Healthy weight	16 (30.2)	30 (56.6)	6 (11.3)	1 (1.90)	15 (28.3)	29 (54.7)	7 (13.2)	1 (1.90)	1 (1.90)	0 (0.00)
Overweight	48 (41.0)	56 (47.9)	12 (10.3)	1 (0.90)	48 (41.0)	54 (46.2)	13 (11.1)	0 (0.00)	1 (0.90)	1 (0.90)
Obesity	40 (44.4)	43 (47.8)	7 (7.80)	0 (0.00)	31 (34.4)	47 (52.2)	10 (11.1)	2 (2.20)	0 (0.00)	0 (0.00)
p-value	0.246				0.621					
Diabetes mellitus										
No diabetes	44 (58.7)	24 (32.0)	5 (6.70)	2 (2.70)	46 (61.3)	22 (29.3)	4 (5.30)	1 (1.30)	1 (1.30)	1 (1.30)
Diabetes	60 (32.3)	105 (56.5)	21 (11.3)	0 (0.00)	48 (25.8)	109 (59.6)	26 (14.0)	2 (1.10)	1 (0.50)	0 (0.00)
p-value	<0.001*				<0.001*					
Hypertension										
No hypertension	41 (57.7)	28 (39.4)	1 (1.40)	1 (1.40)	38 (53.5)	29 (40.8)	3 (4.20)	1 (1.40)	0 (0.00)	0 (0.00)
Hypertension	63 (33.2)	101 (53.2)	25 (13.2)	1 (0.50)	56 (29.5)	102 (53.7)	27 (14.2)	2 (1.10)	2 (1.10)	1 (0.50)
p-value	<0.001*				0.003*					
Diabetes mellitus and hypertension										
No	58 (55.2)	41 (39.0)	5 (4.80)	1 (1.00)	56 (53.3)	41 (39.0)	5 (4.80)	1 (1.00)	1 (1.00)	1 (1.00)
Yes	46 (29.5)	88 (56.4)	21 (13.5)	1 (0.60)	38 (24.4)	90 (57.7)	25 (16.0)	2 (1.30)	1 (0.60)	0 (0.00)
p-value	<0.001*				<0.001*					

Differences in ICA/CCA PSV ratio across the different groups are shown in Table 6. The median ICA/CCA PSV ratio was similar among males and females, smokers and non-smokers and also healthy weight, overweight and obese patients on both sides. Pairwise comparison showed that the median ICA/CCA PSV ratio of patients aged ≤ 50 years was significantly lower than for patients aged 61-70 years on the right and left sides [0.95 (0.79-1.10) vs. 1.10 (0.90-1.30), p = 0.040; 1.00 (0.80-1.24) vs. 1.10 (1.00-1.40), p = 0.032, respectively]. However, the median ICA/CCA PSV ratio was significantly higher among patients with hypertension compared to patients with no hypertension on the right and left sides [1.10 (0.90-1.39) vs. 1.00 (0.86-1.20), p = 0.021; 1.10 (0.90-1.40) vs. 1.00 (0.80-1.22), p = 0.006, respectively] and among patients with diabetes and hypertension compared to patients without both diseases on the right and left sides [1.10 (0.90-1.40) vs. 1.00 (0.87-1.22), p= 0.038; 1.20 (1.00-1.40) vs. 1.00 (0.90-1.20), p= 0.003, respectively].

## Discussion

Significant CAS among patients undergoing coronary artery bypass graft (CABG) increases the risk of a cerebrovascular event [19]. Therefore, a preoperative carotid ultrasound examination is critical to predict perioperative stroke. Carotid ultrasonography is noninvasive, cost effective, and valuable for assessing the carotid artery and improving quality of life [20]. This study aimed to determine the prevalence and common risk factors of CAS among cardiac surgery patients.

In our study, the prevalence of overall CAS among the study population was 71% (n = 187): 52% (n = 136) with bilateral CAS and 19.5% (n =51) with unilateral CAS. The results of our study are consistent with other previous research studies, in which the prevalence of CAS was found to be 77% [21]. Moreover, Zhang et al. (2015) classified the incidence of CAS based on its severity and found 54.5% with mild CAS, 13% with moderate CAS, 4.7% with severe CAS, and 0.8% with occlusion of the carotid artery [15]. These findings are consistent with our study, in which the prevalence of mild (< 50%), moderate (50-69%), severe (>70%), and occlusion was 54.8% (n = 43), 14.6% (n = 38), 1.5% (n = 4), and 1.1% (n = 3), respectively. However, Ranjan et al. (2022) found the overall prevalence of CAS to be 13% lower than the findings in our study. This study contrasts with the aforementioned study, in which there were specific selection criteria and the prevalence of CAS was only found in patients undergoing CABG, with other types of cardiac surgery excluded [11]. Several studies reported the risk factors of significant CAS, such as a history of cerebrovascular accident, diabetes mellitus, hypertension, dyslipidemia, and obesity [10-11, 17]. Our study demonstrates a positive relationship between age and the severity of CAS, with older patients displaying a significant association (p < 0.001). Age group was also significantly associated with bilateral CAS.

This study supports the evidence of a relationship between age and CAS from several previous studies [15,22-23], with the stiffness of the arterial wall increasing with age. However, Adhikary et al. (2019) found that age was not statistically different based on the severity of CAS. This inconsistency may be due to the different study population being examined (patients in Bangladesh). Adhikary et al. (2019) found that 72% were aged 50-59 years, while 28% were aged 60-70 years. Our study found that the majority of patients (83; 79.1%) were aged 61-70 years, while 47 (69.2%) were aged 51-60 years [10].

The results of this study indicate a significant association between hypertension, diabetes mellitus, and a combination of both diseases and CAS (p < 0.05, for all). Furthermore, our study supports evidence that hypertension is another risk factor associated with the severity and bilateral CAS from previous observations, in which high blood pressure leads to the accumulation of cholesterol in the arterial wall, which increases the risk of a plaque rupture [10-11, 15-17, 23]. This study confirms that diabetic mellitus is associated with severity and bilateral CAS. Lu et al. (2020) reported that diabetic mellitus complications affect the microvascular structures, which increase carotid intima media thickness [24]. These results corroborate the findings of numerous previous reports [10-11, 15].

In addition, our study, in contrast to previous studies, found that BMI is not related to the severity and bilateral CAS. This suggests that severe CAS is significantly associated with obesity [10-11, 25]. Another obvious finding to emerge from the analysis is that gender is not associated with the severity or bilateral CAS, which is consistent with other previous studies [10, 25]. However, Zhang et al. (2015) found that being male is one of the independent predictors of severe CAS [15], while Ranjan et al. (2022) found that being female is a potential risk factor associated with significant CAS [11]. These findings do not support our research, but this could be due to the different populations and smaller sample sizes being used in different studies. With that said, this study has several limitations. First, we did not follow up with the patients after their cardiac surgery to evaluate the impact of CAS on perioperative stroke and the outcome of cardiac bypass surgery among patients with CAS. Moreover, ultrasonography is highly operator dependent and the results may be affected by technical factors.

## Conclusions

A high prevalence of CAS was found among patients undergoing cardiac surgery. Age, diabetes mellitus, and hypertension were found to be major risk factors for CAS, as these conditions are significantly associated with the severity and bilateral CAS. Gender and weight status were not associated to CAS. In addition, a preoperative carotid duplex scan is a useful exam to identify CAS among cardiac surgery patients and, therefore, to predict and reduce postoperative neurological complications. Further studies are required to assess the efficacy of ultrasound duplex for evaluating CAS in comparison with CT angiography and for determining the impact of CAS on perioperative stroke.
